# Preparation and Application of a Chemical Probe for Identifying the Targets of the Marine Cyclic Peptide Kapakahine A

**DOI:** 10.3390/molecules27031072

**Published:** 2022-02-05

**Authors:** Rie Kamihira, Yoichi Nakao

**Affiliations:** 1 Research Institute for Science and Engineering, Waseda University, 3-4-1 Okubo, Shinjuku-ku, Tokyo 169-8555, Japan; r-k-cmk@ruri.waseda.jp; 2 School of Advanced Science and Engineering, Waseda University, Shinjuku-ku, Tokyo 169-8555, Japan

**Keywords:** marine natural product, cyclic peptide, chemical fluorescent probe, target protein, chemical biology

## Abstract

Marine organisms are a rich source of bioactive secondary metabolites. Although many marine natural products with bioactivities have been isolated, successful elucidation of their mechanisms of action remains limited. In this study, we prepared a probe molecule based on the marine cyclic peptide kapakahine A (**1**) by introducing a linker with an azide terminal group, which enables the introduction of fluorescent groups for the effective monitoring of subcellular localization, or coupling to affinity beads for the pull-down of target proteins. The results of LC/MS/MS measurements, ProteinPilot analysis, and Western blotting suggest that kapakahine A interacts with the mitochondrial inner membrane proteins PHB1, PHB2, and ANT2, which is consistent with the results of the subcellular localization analysis using a fluorescent probe.

## 1. Introduction

To date, more than 30,000 marine natural products have been reported [1]. To use the potentially unique structures and bioactivities of marine natural products in pharmaceutical materials, it is essential to elucidate their mechanisms of action. Probe molecules based on natural products can be powerful tools in establishing efficient methods of identifying target proteins [2,3]. Fluorescent probes can be used to visualize subcellular localization, and affinity beads can be used to drag out target proteins. However, probe molecules based on natural compounds need to be custom-made because their chemical properties are greatly influenced by the natural compounds themselves, and as a result, they are not always efficiently prepared. If the number of successful target protein identifications can be increased, although it currently remains limited, we may be able to set up a platform for the efficient analysis of the mechanisms of action of marine natural compounds by integrating the methods developed during the process.

Cyclic peptides are one of the major categories of marine natural products, and many marine cyclic peptides with various bioactivities have been reported to date [4,5,6]. Among them, aurilide—which exhibits strong cytotoxicity—is an example for which the target protein has been successfully identified as prohibitin 1 (PHB1) in mitochondria. The mechanism of action of this compound has been described as the binding of the molecule to PHB1, which leads to mitochondrial fragmentation and the induction of cell apoptosis [7]. In our continuing study, we are focused on kapakahines, a family of marine cyclic peptides isolated from the sponge *Cribrochalina olemda*. Kapakahines share a unique structural motif in which the nitrogen in the indole ring of the *N*-terminal tryptophan residue is directly attached to a tetracyclic core containing an α-carboline skeleton. Kapakahine A is the main compound in this family and is cytotoxic to the P388 murine myeloid leukemia cell line in the order of µM and has an *N*-terminal reactive amino group that readily binds to active esters [8]. Compounds such as aurilide—which has cytotoxicity in the order of nM because of its high affinity for the target protein—are generally considered suitable for target identification by means of chemical probes attached to beads. However, if the same approach could be made applicable to the identification of the target proteins of compounds with a weaker cytotoxicity in the order of µM such as kapakahine A, it would pave the way for the mechanistic analysis of many marine natural products that do not have potent activity.

Rocha et al. synthesized a fluorescent probe comprising kapakahine E conjugated with coumarin and found that the fluorescent probe localized to the Golgi apparatus in PC-3M prostate carcinoma cells [9]. In a previous study, we prepared a fluorescent probe of kapakahine A [Kap A-5FL (**2**)] by direct coupling with an FITC derivative with an active ester linker [10]. Co-localization analysis of Kap A-5FL (**2**) and Golgi-binding lectin HPA, or concanavalin A (endoplasmic reticulum marker) in HeLa cells, showed that Kap A-5FL does not localize in the Golgi apparatus or endoplasmic reticulum.

In this study, we describe the preparation of Kap A-beads (**6**) as well as Kap A-5FL localization with organelles in P388 cells and the target protein candidates of kapakahine A (**1**).

## 2. Results

### 2.1. Preparation of the Linker-Conjugated Probe (**3**) and Kapakahine A Beads (**6**)

**Figure 1:**
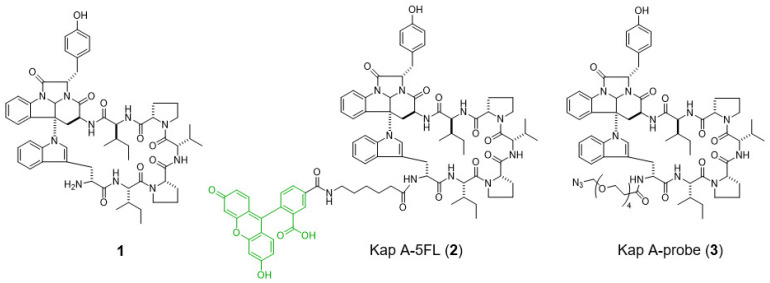
Structures of kapakahine A (**1**), fluorescently labeled kapakahine A (**2**), and the linker-conjugated probe (**3**).

**Scheme 1:**
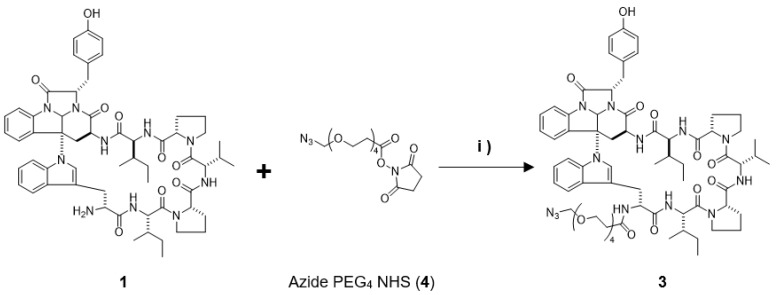
The reaction conditions for preparing the linker-conjugated probe **3**. (i) DMF, excess **4**, rt, 20 h.

**Scheme 2:**
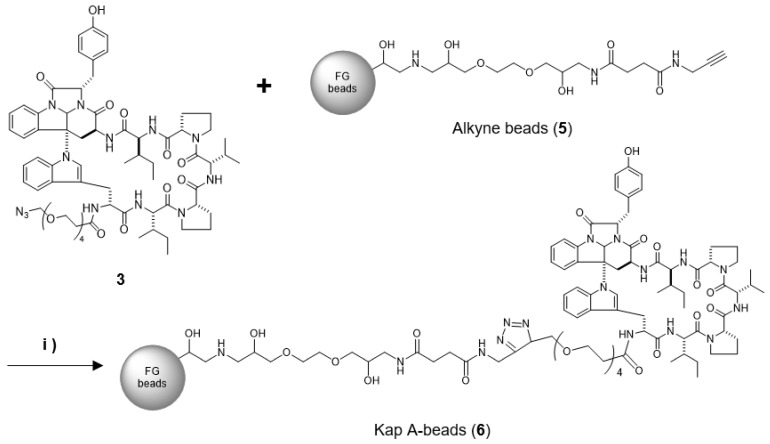
The reaction conditions for preparing Kap A-beads (**6**) using a click reaction. (i) *^t^*BuOH/DMSO/H_2_O (4/1/5), TBTA, L-ascorbic acid, CuSO_4_ aq., rt, 20 h.

To prepare the linker-conjugated probe based on kapakahine A (**1**, Figure 1), we modified the *N*-terminal amino group of **1** with the linker reagent, azide-PEG_4_-NHS (**4**). Reagent **4** has a highly reactive *N*-hydroxysuccinimide (NHS) group (Scheme 1) that can react with the amino group of kapakahine A under mild conditions. The conjugation of **4** and kapakahine A (**1**, 1.0 mg) was carried out and the reaction mixture was subjected to HPLC (high-performance liquid chromatography) purification giving the linker-conjugated probe (**3**, 0.5 mg, Scheme 1). The prepared linker-conjugated probe (**3**, 0.1 mg) was then coupled to functional magnetic beads (FG beads, **5**, 1.0 mg) via a “click reaction” between the azide group of the probe and the alkyne group of the FG beads, to yield Kap A-beads (**6**, Scheme 2).

### 2.2. Visualization of Subcellular Localization

**Figure 2:**
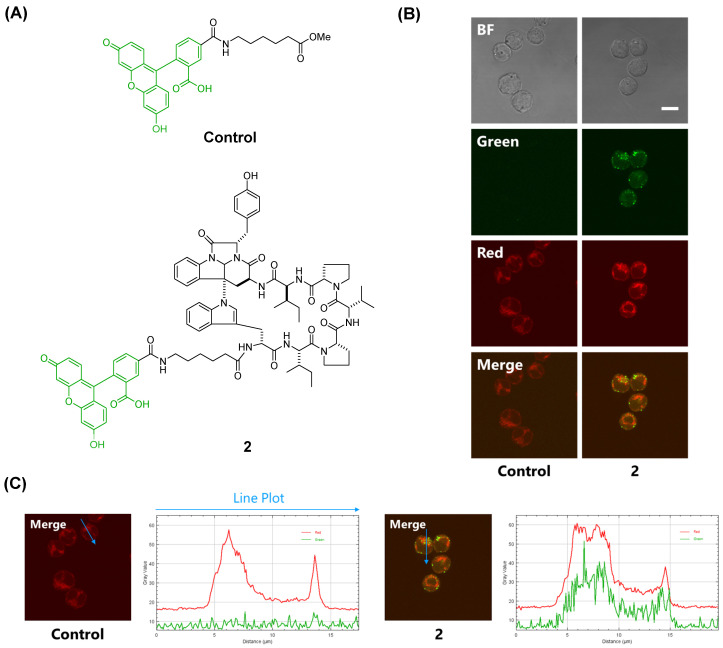
Co-localization studies in P388 cells. (**A**) Structures of the fluorescent probes. (**B**) Confocal microscopy images of P388 cells incubated with **2** with Lipofectamine 2000 for 2 h (green). During the incubation, the live cells were stained with MitoTracker (red). Magnification ×100, scale bar: 20 μm. The brightness and contrast were equally adjusted for all the green and red fluorescence images. (**C**) Co-localization analysis of P388 cells. The line profile plots analysis using Image J showed the green and red fluorescence intensity distribution through the blue line.

In our previous report [10], the fluorescently labeled kapakahine A in HeLa cells was not found to subcellularly localize with the endoplasmic reticulum or the Golgi apparatus; however, a fluorescent probe of kapakahine E was shown to localize with the Golgi apparatus [3]. To investigate the subcellular localization of the kapakahine A fluorescent probe in P388 cells—towards which kapakahine A shows cytotoxicity—co-staining with Kap A-5FL (**2**) and MitoTracker was carried out. The fluorescently-labeled Kap A-5FL (**2**) was prepared as previously described [4], and was administered using Lipofectamine 2000 to P388 cells seeded in a 96-well plate at a concentration of 20 µM prior to the addition of MitoTracker. Confocal microscopy images showed that the green fluorescence of Kap A-5FL (**2**) was co-localized within the mitochondria (Figure 2).

### 2.3. Determining the Target Proteins of Kapakahine A

**Figure 3:**
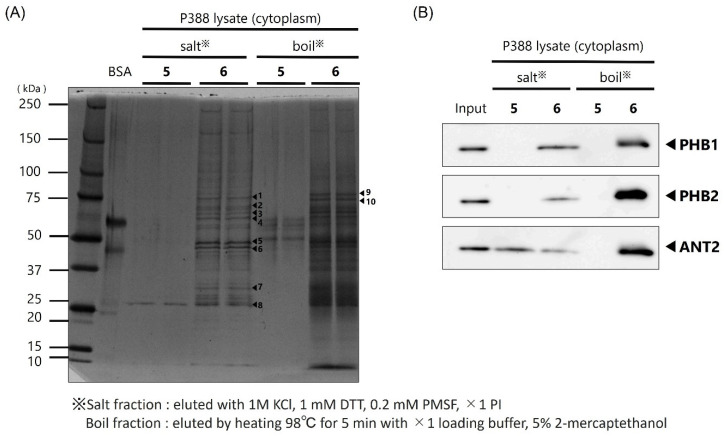
Identification of the binding proteins of kapakahine A. (**A**) Protein bands specifically bound to Kap A-beads (**6**) were pulled down from the cytoplasmic protein extract of P388 cells and detected by silver staining. Alkyne beads (**5**) were used as the control. The proteins contained in each band were identified by LC/MS/MS analysis. (**B**) Western blotting with antibodies against target protein candidates. PHB1, PHB2, and ANT2 were identified as binding proteins.

To investigate the target proteins of kapakahine A, we performed pull-down assays using Kap A-beads (**6**) and cytoplasmic fraction prepared from P388 cell extracts based on the subcellular localization of kapakahine A in cytoplasm. The Kap A-beads (**6**) and the cytoplasmic fraction were incubated at 4 °C for 4 h; then, after the beads were washed, the bound proteins were collected from the beads as salt-eluted and heat-eluted fractions. The fractions were subjected to SDS-PAGE, and 10 bands specific to the Kap A beads (**6**) were cut out of the gels and analyzed by LC/MS/MS (Figure 3A). The results identified six mitochondrial proteins as candidate target (see Table 1). Among them, the mitochondrial inner membrane proteins prohibitin 1 (PHB1), prohibitin 2 (PHB2), and ADP/ATP translocase 2 (ANT2) were further confirmed by Western blotting (Figure 3B).

## 3. Discussion

In this study, we prepared a linker-conjugated probe, Kap A-probe (**3**), based on the marine cyclic peptide kapakahine A (**1**). A pull-down assay using Kap A-beads (**6**) identified PHB1, PHB2, and ANT2 as target protein candidates. These candidates are all mitochondrial proteins, which is consistent with the finding that the kapakahine A fluorescent probe co-localized with the mitochondria.

PHB1 is known to be the target protein of aurilide, a nanometer-order cytotoxic marine cyclic peptide that induces mitochondrial fragmentation and apoptosis by forming a complex with PHB1 that releases m-AAA involved in the processing of L-OPA1 to S-OPA1 [7]. PHBs are known to be multifunctional proteins in mitochondria [11,12,13]. It has recently been suggested that PHB2 and ANT2 regulate the uptake of substances such as ATP by forming mitochondrial transmembrane pores, and play an important role in mitophagy, which is involved in maintaining mitochondrial quality [14,15].

Sesaminol, an ingredient of sesame that is cytotoxic towards the MCF7 human mammary carcinoma cell line [16], is known to bind to ANT2 and reduce the protein expression of cyclin D1, which is important for the G_1_/S phase transition of the cell cycle. Rabdosianone I, from the medicinal herb *Isodon japonicus*, binds to PHB2 and ANT2, leading to a decrease in the expression level of thymidylate synthase, resulting in growth inhibition of the human colon adenocarcinoma cell line HT-29 [17].

In this study, we successfully prepared Kap A-beads (**6**) and detected three target protein candidates: PHB1, PHB2, and ANT2. These proteins are known to localize in mitochondria, which is where kapakahine A has been shown to localize. The cytotoxicity mechanisms of sesaminol and rabdosianone I target ANT2 or PHB2; therefore, the cytotoxicity mechanism of action of kapakahine A may be explained by its binding to ANT2 or PHB2. The possibility that PHB1 is a target molecule of kapakahine A can potentially be excluded because the cytotoxicity of kapakahine A was much weaker than that of aurilide and the fragmentation of mitochondria was not observed following the addition of kapakahine A.

Although further study is needed to confirm the mechanism of action of kapakahine A, it was shown that useful mechanistic information can be obtained for moderately active natural products using naturally available quantities.

## 4. Materials and Methods

### 4.1. Materials

The confocal microscopy images were acquired using an Olympus FV1000 (Olympus Corporation, Tokyo, Japan). Nuclear magnetic resonance (NMR) spectra were measured with a Bruker Avance (600 MHz, Bruker Corporation, Billerica, MA, USA) spectrometer. HPLC (high-performance liquid chromatography) was conducted using a JASCO PU-1580 pump system equipped with TOSOH UV8011 (TOSOH Corporation, Tokyo, Japan).

All high-resolution mass spectra (HRMS) were recorded on a JEOL JMS-T100CS spectrometer (JEOL Ltd., Tokyo, Japan) at the Materials Characterization Central Laboratory, Waseda University. LC/MS/MS was performed using a Triple TOF 4600 system (AB Sciex, Tokyo, Japan).

### 4.2. Preparation of the Kap A-Probe (**3**)

Kapakahine A (**1**, 1.0 mg, 0.95 µmol) dissolved in 100 µL of DMF was mixed with 3 µL (9.0 µmol) of compound **4** and allowed to react for 20 h, stirring at room temperature while shielded from light. The collected reaction mixture was concentrated and dried, then subjected to reversed-phase HPLC [COSMOSIL 5C_18_-AR II (φ10 × 250 mm), flow rate; 2 mL/min, isocratic elution with 55% acetonitrile, detection; 220 nm JASCO] yielding Kap A-probe (**3**, 0.5 mg), which showed a [M-H]^−^ ion peak at *m*/*z* 1324.6726 (calcd. C_69_H_90_N_13_O_14_ 1324.6736). The mass spectrum (Figure S1), ^1^H-NMR spectrum (Figure S2), and HPLC chart (Figure S3) are shown in Supplementary Materials.

### 4.3. Cell Culture

Roswell Park Memorial Institute medium (RPMI, FUJIFILM Wako Pure Chemical Corporation, Osaka, Japan) supplemented with HEDS solution (2,20-dithiobisethanol) and kanamycin sulfate was used as the culture media for P388 murine leukemia cells. Cells were cultured at 37 °C under an atmosphere of 5% CO_2_.

### 4.4. Staining of P388 Cells

A fluorescent control and fluorescently labeled kapakahine A (**2**) were prepared as previously described [4]. P388 cells (10,000 cells/well in 200 µL medium) were plated in each well of a 96-well glass bottom microplate. After incubation for 24 h, the cultural medium in each well was removed and replaced with 100 µL of a solution of fluorescent probe **2** prepared with Lipofectamine 2000 [4]; then, cells were further incubated at 37 °C under an atmosphere of 5% CO_2_ for 2 h. The concentration of compound **2** was 20 μM. After 75 min, 1 mg/mL of MitoTracker Red CMXRos (Thermo Fisher Scientific Inc., Waltham, MA, USA) dissolved in DMSO was diluted with culture medium and 11 µL of the mixture was added to the cells and incubated at 37 °C under an atmosphere of 5% CO_2_ for 15 min. The cells were fixed in 100 µL of 4% paraformaldehyde at 37 °C under an atmosphere of 5% CO_2_ for 15 min. After fixing and washing with culture medium and PBS, fluorescence cell images were acquired at ×100 magnification using a confocal microscope system.

### 4.5. Preparation of Protein Extracts Containing Cytoplasmic and Plasma Membrane Fractions of P388 Cells

P388 cells that had reached confluency were collected in 15 mL tubes and centrifuged at 180× *g* for 5 min at 15 °C. Cold PBS (1 mL) was added to the precipitate obtained by removing the supernatant, then transferred to a 2.0 mL tube and centrifuged at 1500× *g* for 5 min. This washing procedure was carried out again, and 200 µL of buffer A [10 mM HEPES-NaOH (pH 7.9), 10 mM KCl, 0.1 mM EDTA, 1 mM DTT, 0.5 mM PMSF (phenylmethylsulfonyl fluoride), ×1 protease inhibitor cocktail (PI)] was added to the precipitate. After standing on ice for 15 min, 11 µL of 10% NP-40 was added and cells were agitated. The supernatant was then collected following centrifugation at 215× *g* for 5 min. The supernatant was diluted with buffer B [20 mM HEPES-NaOH (pH 7.9), 100 mM KCl, 1 mM MgCl_2_, 0.2 mM CaCl_2_, 0.2 mM EDTA, 10% (*v*/*v*) glycerol, 1 mM DTT, 0.5 mM PMSF, ×1 PI] to ensure a 0.1% NP-40 concentration to yield protein extracts including cytoplasmic and plasma membrane fractions.

### 4.6. Preparation of Kap A-Beads (**6**)

Magnetic FG beads (**5**, 1.0 mg) with terminal alkynes (Tamagawa, Seiki Co., Ltd., Nagano, Japan) were mixed with 25 µL of Kap A-probe (**3**) prepared at 1 mM in ^*t*^BuOH/DMSO (4/1) and 25 µL of 500 µM TBTA, then sonicated. To this solution, 25 µL each of 5 mM aqueous CuSO_4_ solution and 5 mM aqueous sodium L-ascorbate solution were added, and the reaction was allowed to proceed at room temperature for 20 h, being shaken by a microtube mixer. The reaction solution was centrifuged at 18,800× *g* for 5 min, and then the supernatant was collected. The precipitated beads were washed with 200 µL of ^t^BuOH/DMSO/H_2_O (4/1/2) and centrifuged at 18,800× *g* for 5 min, followed by washing to remove the supernatant three times. The washing operation was then repeated three times with 200 µL of 50% MeOH. Finally, 40 µL of 50% MeOH was added to yield Kap A-beads (**6**).

### 4.7. Binding of Kap A-Beads (**6**) to Proteins

Protein extracts containing cytoplasmic and membrane fractions from P388 cells were mixed with binding buffer (20 mM HEPES-NaOH, pH 7.9, 100 mM KCl, 1 mM MgCl_2_, 0.2 mM CaCl_2_, 0.2 mM EDTA, 10% glycerol, 0.1% NP-40, 1 mM DTT, 0.2 mM PMSF, ×1 PI) which was used to dispense 200 µg. After centrifugation at 215× *g* for 30 min at 4 °C, the supernatant was mixed with 0.5 mg of Kap A-beads (**6**). Alkyne beads (**5**) were used as a control. The mixed solution of supernatant and FG beads was rotated at 4 °C for 4 h to allow the binding reaction to proceed. After the reaction, the mixture was subjected to magnetic separation for 5 min and the supernatant was discarded. Binding buffer (200 µL) was added to the remaining beads, which were then spun down and subjected to magnetic separation for 5 min, and the supernatant was discarded. This washing procedure was repeated twice more. Salt elution buffer (30 µL; 1 M KCl, 1 mM DTT, 0.2 mM PMSF, ×1 PI) was added to the washed beads, and the beads were dispersed and placed on ice for 5 min. After magnetic separation, the supernatant was collected in a 1.5 mL tube and mixed with 8 µL of ×4 loading buffer (0.25 mM Tris-HCl pH 6.8, 0.02% bromophenol blue, 8% SDS, 40% glycerol) and 2 µL of 2-mercaptoethanol, and then heated at 98 °C for 5 min to obtain the salt-eluted bound protein fraction. The remaining beads were dispersed by adding 38 µL of ×1 loading buffer and 2 µL of 2-mercaptoethanol, and then heated at 98 °C for 5 min to obtain the heat-eluted bound protein fraction.

The obtained protein solutions were subjected to SDS-PAGE using Super Sep™ Ace, 5–20% gradient gel (FUJIFILM Wako Pure Chemical Corporation, Osaka, Japan) with 25 mM Tris, 192 mM glycine, and 0.1% SDS as the buffer for 90 min at a constant current of 18 mA. Precision Plus Protein Dual Color Standards (Bio-Rad, Hercules, CA, USA) were used as molecular weight markers. Protein bands on the gel were detected by silver staining according to the manufacturer’s protocol.

### 4.8. Target Protein Identification

The protein bands of interest were cut out of the gel after silver staining and collected in 1.5 mL tubes. Decolorizing solution (100 µL) was added, and the samples were allowed to stand for 15 min. The supernatant was discarded, and washing was repeated twice with water. Acetonitrile (100%, 200 µL) was then added and allowed to stand for 5 min, and the gel fragments were dried using a lyophilizer. Two hundred microliters of 10 mM DTT, 25 mM NH_4_HCO_3_ mixed solution was added to the dried gel and the proteins were reduced by heating at 56 °C for 45 min. After discarding the solution, 200 µL of 25 mM NH_4_HCO_3_ solution was added, the sample was shaken for 5 min, and the solution removal was repeated twice. Acetonitrile (100%, 200 µL) was then added and allowed to stand for 5 min, and the gel fragments were dried using a lyophilizer. Trypsin (10 µL, Trypsin Gold, Mass Spectrometry Grade, Promega, WI, USA) was added to 10 µg/mL of the dried gel sections, and digestion was allowed to proceed in the gel at 37 °C for 18 h. Acetonitrile (50%) containing 0.1% TFA (trifluoro acetic acid) was added, and the solution was collected following sonication. This procedure was repeated twice, and the recovered solution was concentrated for LC/MS measurement. The LC/MS data were analyzed using ProteinPilot Software 4.5 (AB Sciex, Tokyo, Japan) for the identification of proteins.

### 4.9. Western Blotting

After separation of the protein bands by SDS-PAGE, the gel was fixed in 100% MeOH. The gel was then immersed in transfer buffer (tris-hydroxyaminomethane 25 mM, glycine 192 mM, 20% MeOH) and the proteins in the gel were transferred to a polyvinylidene fluoride (PVDF) membrane using a transfer system at a constant current of 180 mA for 60 min. The transferred membranes were fixed and stained with Ponceau stain and then decolorized. Blocking was then carried out using 10% skim milk powder dissolved in TBS-T for 60 min at room temperature. Then, primary antibodies anti-prohibitin 1 (1:100, sc-377037, Santa Cruz Biotechnology, Inc., Dallas, TX, USA), anti-prohibitin 2 (1:100, sc-133094; Santa Cruz Biotechnology, Inc., Dallas, TX, USA), and anti-ANT2 (1:1000, ANT2/SLC25A5(E2B9D), Cell Signaling Technology, Danvers, MA, USA), diluted in PBS-T solution containing 1% skim milk, were allowed to react overnight at 4 °C. Shaking and washing with PBS-T for 5 min was performed three times; then, the antibodies were reacted with secondary antibodies for 3 h at room temperature. After 3 rounds of shaking and washing with PBS-T for 5 min, protein detection was performed using SuperSignal West Pico Chemiluminescent Substrate or Super Signal West Femto Maximum Sensitivity Substrate (Bio-Rad, Hercules, CA, USA). An LAS-3000 (FUJIFILM, Tokyo, Japan) was used to photograph the membrane.

## Figures and Tables

**Table 1:** Kapakahine A target protein candidates identified from P388 cells.

Band Number	Target Protein Candidates Identified by ProteinPilot Analysis
No. 1	Not detected.
No. 2	Not detected.
No. 3	REVERSED SHC transforming protein 1 cytoplasmic and mitochondrion matrix
No. 4	Not detected.
No. 5	ATP synthase subunit α
No. 6	ATP synthase subunit β
No. 7	Prohibitin 2 (PHB2)
No. 8	ADP/ATP translocase 2 (ANT2), prohibitin 1 (PHB1)
No. 9	REVERSED solute carrier family 12 member 1
No. 10	Mitochondrial inner membrane protein (Mic60, mitofilin)

## Data Availability

Not applicable.

## Supplementary Materials

The following supporting information can be downloaded. Figure S1: HRESIMS (neg.) of Kap A-probe (**3**), Figure S2: ^1^H NMR spectrum (600 MHz, methanol-*d*_4_) of Kap A-probe (**3**), Figure S3: HPLC chart of Kap A-probe (**3**).

## Sample Availability

Samples of the compounds are available from the authors.
