# Supplementary material for: Preparation and Application of a Chemical Probe for Identifying the Targets of the Marine Cyclic Peptide Kapakahine A
Source: Molecules. 2022 Feb 5;27(3):1072. doi: 10.3390/molecules27031072 (PMC8838629; doi:10.3390/molecules27031072)
Supplement: Supplementary file 1 [file molecules-27-01072-s001.zip › molecules-1575592-supplementary.pdf]

Supporting Information

Preparation and Application of a Chemical Probe for Identifying the Targets of the Marine Cyclic Peptide Kapakahine A

Rie Kamihira [1], Yoichi Nakao [1,2,*]

[1] Research Institute for Science and Engineering, Waseda University, 3-4-1 Okubo, Shinjuku-ku, Tokyo 169-8555, Japan

[2] School of Advanced Science and Engineering, Waseda University, Shinjuku-ku, Tokyo 169-8555, Japan

* Correspondence: ayocha@waseda.jp; Tel.: +81-3-5286-3100

Table of contents

Experimental section

Figure S1. HRESIMS (neg.) of Kap A-probe (**3**).

Figure S2. ^{1}H NMR spectrum (600 MHz, methanol-d_4) of Kap A-probe (**3**). **Figure S3**. HPLC chart of Kap A-probe (**3**).

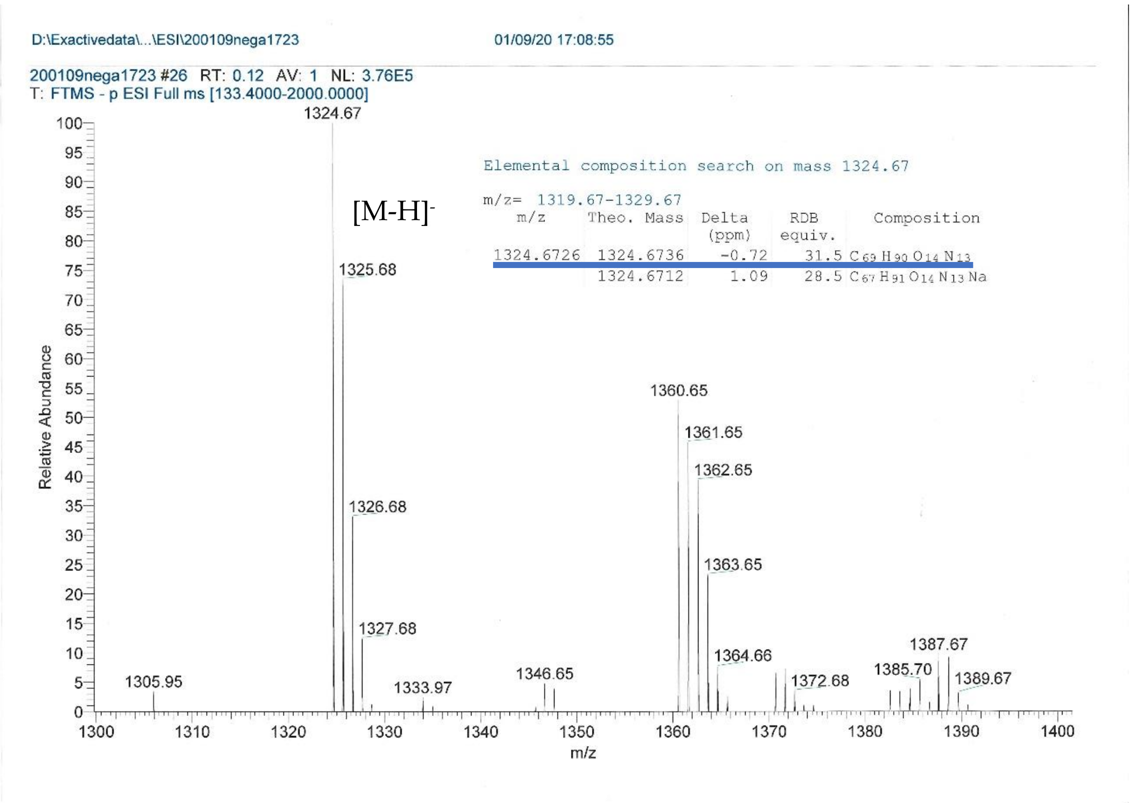

Figure S1. HRESIMS (neg.) of Kap A-probe (**3**).

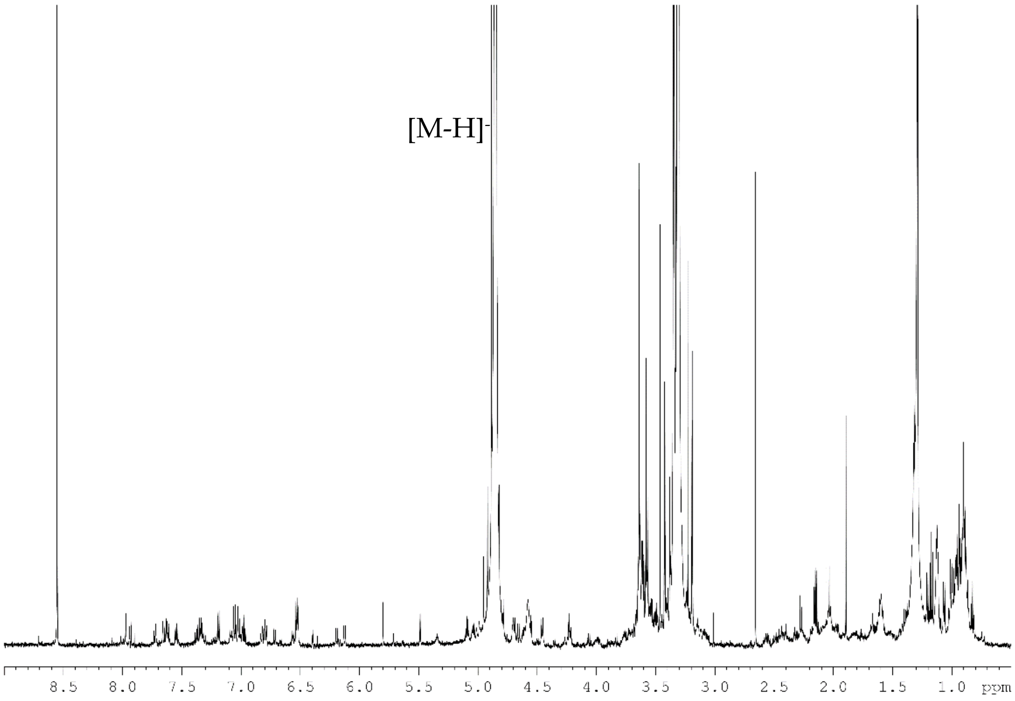

Figure S2. ^{1}H NMR spectrum (600 MHz, methanol-d_4) of Kap A-probe (**3**).

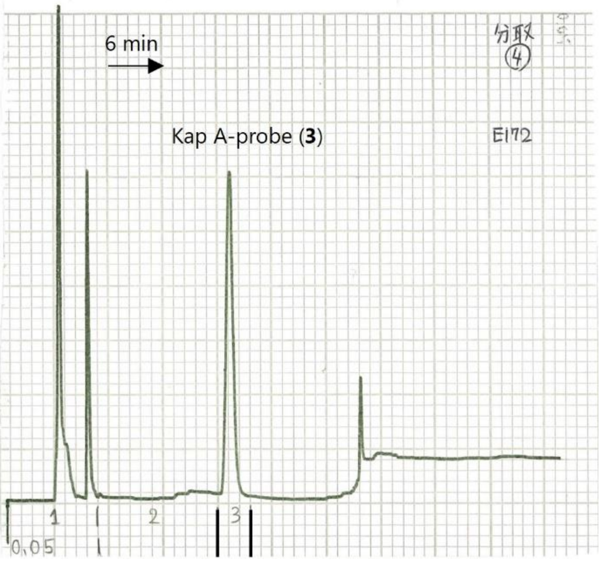

Figure S3. HPLC chart of Kap A probe (**3**).
